# Live cell screening identifies glycosides as enhancers of cardiomyocyte cell cycle activity

**DOI:** 10.3389/fcvm.2022.901396

**Published:** 2022-09-26

**Authors:** Ajit Magadum, Harsha V. Renikunta, Neha Singh, Conchi Estaras, Raj Kishore, Felix B. Engel

**Affiliations:** ^1^Department of Cardiac Development and Remodelling, Max-Planck-Institute for Heart and Lung Research, Bad Nauheim, Germany; ^2^Cardiovascular Research Center, Icahn School of Medicine at Mount Sinai, New York, NY, United States; ^3^Lewis Katz School of Medicine, Center for Translational Medicine, Temple University, Philadelphia, PA, United States; ^4^Department of Cardiology, Charité Berlin - University Medicine, Berlin, Germany; ^5^Department of Sports Biosciences, Central University of Rajasthan, Ajmer, India; ^6^Department of Cardiovascular Sciences, Lewis Katz School of Medicine at Temple University, Philadelphia, PA, United States; ^7^Experimental Renal and Cardiovascular Research, Department of Nephropathology, Institute of Pathology, Friedrich-Alexander-Universität Erlangen-Nürnberg (FAU), Erlangen, Germany; ^8^Muscle Research Center Erlangen (MURCE), Erlangen, Germany

**Keywords:** cardiac glycosides, cardiomyocyte proliferation, calcium handling, live cell screening platform AG, Azami Green, stem cell, small molecules, cell cycle

## Abstract

Promoting cardiomyocyte proliferation is a promising strategy to regenerate the heart. Yet, so far, it is poorly understood how cardiomyocyte proliferation is regulated, and no factor identified to promote mammalian cardiomyocyte proliferation has been translated into medical practice. Therefore, finding a novel factor will be vital. Here, we established a live cell screening based on mouse embryonic stem cell-derived cardiomyocytes expressing a non-functional human geminin deletion mutant fused to Azami Green (CM7/1-hgem-derived cardiomyocytes). We screened for a subset of compounds of the small molecule library Spectrum Collection and identified 19 potential inducers of stem cell-derived cardiomyocyte proliferation. Furthermore, the pro-proliferative potential of identified candidate compounds was validated in neonatal and adult rat cardiomyocytes as well as human induced pluripotent stem cell-derived cardiomyocytes. 18 of these compounds promoted mitosis and cytokinesis in neonatal rat cardiomyocytes. Among the top four candidates were two cardiac glycosides, peruvoside and convallatoxin, the flavonoid osajin, and the selective α-adrenoceptor antagonist and imidazoline I1 receptor ligand efaroxan hydrochloride. Inhibition of PTEN and GSK-3β enhanced cell cycle re-entry and progression upon stimulation with cardiac glycosides and osajin, while inhibition of IP3 receptors inhibited the cell cycle-promoting effect of cardiac glycosides. Collectively, we established a screening system and identified potential compounds to promote cardiomyocyte proliferation. Our data suggest that modulation of calcium handling and metabolism promotes cardiomyocyte proliferation, and cardiac glycosides might, besides increasing myocardial contraction force, contribute to cardiac repair by inducing cardiomyocyte proliferation.

## Introduction

Heart failure remains a major socio-economic challenge. Despite significant achievements in medical practice resulting in reduced acute mortality of myocardial infarction (MI), the prevalence of heart failure is increasing (1). Thus, there is a great need to develop strategies that allow to increase the muscle mass of the heart to enhance heart function. In recent years, induction of cardiomyocyte proliferation has been proposed as an important approach for cardiac regeneration (2). An alternative is cardiac tissue engineering. Also in this context, induction of proliferation of stem cell-derived cardiomyocytes is of interest, for example, to enable the generation of compact cardiac tissues during biofabrication (3–5).

Human cardiac regeneration is limited due to low postnatal cardiomyocyte replicative rates as well as progressive polyploidization (2, 6). The mechanism underlying the establishment of the cell cycle arrest in mammalian cardiomyocytes remains poorly understood. Recently, several mechanisms have been suggested including sarcomere formation (7–9), cyclin G1 expression (10), heterochromatin formation (11), loss of centrosome integrity (12), as well as metabolic switch (13, 14). In addition, while initially very few factors were identified to induce significant cardiomyocyte proliferation, in the last decade, a large number of stimuli have been reported to induce cardiomyocyte proliferation and heart regeneration (2, 15–17). Yet, none of these factors has so far been translated into medical practice.

Here, we have applied a chemical approach to modulate proliferation of stem cell-derived cardiomyocytes, which has several advantages over conventional genetic methods such as enabling temporal control, rapid inhibition or activation, regulation of functionally overlapping targets, and applicability of the identified chemicals across similar species. Notably, many chemicals can be applied directly as therapeutic drugs. In addition, we have established a Fucci-based (18) live cell screening, which eliminates the need for techniques such as immunofluorescence staining, incorporation of nucleotide analogs, or cell count assays. In addition, a live cell screening can capture events that may develop at different times post-treatment, which may be potentially overlooked by end-point assays. Screening of over 700 compounds and validation in primary cardiomyocytes identified as the four most potent promotors of cardiomyocyte cell cycle progression two cardiac glycosides, peruvoside and convallatoxin, the flavonoid osajin, and the selective α-adrenoceptor antagonist and imidazoline I1 receptor ligand efaroxan hydrochloride. To date, no study reports that any of these four compounds have an effect on cardiomyocyte cell cycle progression or heart regeneration.

Cardiac glycosides are a group of compounds, which are secondary metabolites produced by certain plants, insects, and vertebrates. They are mainly known as inhibitors of the sodium-potassium pump in eukaryotic cells and are used as drugs to treat heart disease (e.g., cardiac arrhythmia, congestive heart failure, and atrial fibrillation) by increasing myocardial contraction force and, at the same time, lowering the frequency of this contraction (19–21). Interestingly, data are accumulating which indicate that cardiac glycosides have additional targets such as the nuclear receptor superfamily of transcription factors (19). However, their toxicity prevents their widespread use, and thus a better understanding of the function of cardiac glycosides is necessary (20).

## Methods

### Isolation of primary cardiomyocytes

The investigation conforms with the guidelines from Directive 2010/63/EU of the European Parliament on the protection of animals used for scientific purposes. Extraction of organs and preparation of primary cell cultures were approved by the local Animal Ethics Committee in accordance with governmental and international guidelines on animal experimentation (protocol TS-9/2016 Nephropatho). Ventricular cardiomyocytes from 3-day-old (P3) and 12-week-old (adult) Sprague Dawley rats were isolated and cultured as described (22). Rats were first injected s.c. with 0.04 mg/kg buprenorphine and were anesthetized after 30 min by isoflurane inhalation (2 ml vaporized in a 5 l beaker). After loss of standing, eyelid and pedal reflexes, animals were sacrificed by exsanguination due to heart excision upon thoracotomy. Hearts from P3 rats were dissected upon decapitation with operating scissors (ROBOZ [RS-6845], no anesthesia), base with atria removed, and the remaining ventricle minced. Cells were initially cultured for 48–72 h in the presence of 20 μM cytosine-D-arabinofuranoside (ara C) and 5% horse serum before stimulation to prevent non-myocyte proliferation. Cardiomyocytes were subsequently treated in the presence of fetal bovine serum (FBS) (neonatal: 0.2% FBS; adult: 0.5% FBS: adult) as indicated. Postnatal cardiomyocytes were stimulated once; adult cardiomyocytes every day.

### Reagents

The chemical reagents and recombinant proteins were obtained from different companies: “Spectrum Collection” (MicroSource Discovery Systems, Inc., 10 mM stock solutions in dimethylsulfoxid (DMSO), purity > 90%), 6-bromoindirubin-3'-oxime (BIO), osajin, peruvotoxin, efaroxan hydrochloride, xestospongin C (Tocris bioscience), SB203580 (Calbiochem), fibroblast growth factor 1 (FGF1, R&D Systems), convallatoxin (Sigma), valeryl salicylate, bpV(HOpic) (Santa Cruz Biotechnology). Inhibitors were added 1 h before cardiomyocyte stimulation.

### Generation of CM7/1-hgem mouse stem cell line

The mouse embryonic stem (ES) cell line CM7/1 (23) was cultured in stem cell medium [DMEM containing 2 mM L-glutamine (GIBCO), penicillin (100 U/mL), streptomycin (100 μg/mL) (Sigma), beta-mercaptoethanol (Merck), 3 mM Na-pyruvate (Thermo Fisher), and 15% FBS (PAA Laboratories)] for 2 days in the presence of leukemia inhibitory factor (LIF, Sigma). The cells were transfected with linear mAG-hGem (1/110) plasmid (18) using lipofectamine 2000 (Thermo Fisher). After 48 h, cells were selected for utilizing 200 μM Zeocin antibiotic (Thermo Fisher) for 2 weeks. Single growing colonies were selected and expanded, resulting in several cell lines. One of the cell lines showing Azami Green (AG) expression was differentiated into beating embryoid bodies (Ebs) and then dissociated into cardiomyocytes. This mouse stem cell line was called CM7/1-hgem and was used in this study.

### Differentiation of CM7/1-hgem mouse stem cell line

CM7/1-hgem ES cells were cultured in stem cell medium for 2 days in the presence of LIF (Sigma). The cells were trypsinized with 0.005% trypsin (GIBCO) and counted by hemocytometer. For differentiation, 330,000 cells/ 10 ml differentiation medium (Dulbecco's Modified Eagle Medium (DMEM) containing 2 mM L-glutamine, penicillin (100 U/mL), streptomycin (100 μg/mL), and 3 mM Na-pyruvate, 10% FBS) were used for the hanging drop method. After 2 days, formed Ebs were transferred to suspension culture (10 cm cell culture plate) containing differentiation medium. The plates were stirred at 50 rpm. On day 9, when Ebs started to beat, differentiation medium was replaced with differentiation medium containing 400 μM G418 (Thermo Fisher) to eliminate non-myocytes. After 4–5 days, Ebs were dissociated with 1 mM collagenase B (Sigma) in phosphate-buffered saline (PBS) and centrifuged at 400 RCF for 5 min. Subsequently, single cells were seeded on fibronectin-coated cell culture plates (15,000 cells/well) in cardiomyocyte medium (DMEM containing 2 mM L-glutamine, penicillin (100 U/mL), streptomycin (100 μg/mL), and 3 mM Na-pyruvate, 0.2% FBS) and 400 μM G418 (100 μl/well) and cultured for 5 to 6 days. Then, cells were washed and treated with small molecules utilizing a cardiomyocyte medium.

### Screening

CM7/1-hgem-derived cells were treated once with the indicated compounds at a concentration of 1 μM. More than 700 molecules were screened from the bioactive collection of the “Spectrum Collection” from MicroSource Discovery Systems, Inc. (Gaylordsville, CT) (Supplementary Table 1). We used cardiomyocyte medium with DMSO as negative control and 10% FBS and FGF1 + p38 mitogen-activated protein kinase inhibitor SB203580 (FGF1/p38i) as positive controls. AG expression was analyzed every 12 h (for quantitative analysis, around 100 cells were evaluated) for the following 4 days by visual inspection using a Leica fluorescence microscope. The maximal number of mAG-hGem(1/110)-positive cells were used to normalize the data against the DMSO-treated control as a fold-change. Hit compounds were defined as those giving an effect higher than 2-fold. Individual samples of hit compounds were picked from the original library and confirmed with the same method as in the primary screen for three times.

### Reverse transcription followed by polymerase chain reaction (RT-PCR) analysis

Total ribonucleic acid (RNA) was isolated from Ebs derived from CM7/1-hgem mouse stem cells at different time points using the Rneasy Kit (Qiagen). The cDNA (complementary deoxyribonucleic acid) was prepared from isolated RNA using *MML*V or superscript II reverse transcriptase and the oligo(dT) primer (Qiagen). PCR was performed according to standard protocols (Applied Biosystems). Primers: *octamer binding transcription factor* (*oct*)*3/4*: forward (F) 5′- TGAGAACCTTCAGGAGATATGCAA−3′, reverse ©, 5′- CTCAATGCTAGTTCGCTTTCTCTTC−3′, *nanog*: F 5′-AGTATCCCAGCATCCATTGC−3′, R, 5′- TTTCACCTGGTGGAGTCACA−3′, *brachyury*: F 5′-CTCCAACCTATGCGGACAAT−3′, R, 5′- CCCCTTCATACATCGGAGAA−3′, *isl1*: F 5′- GCGACATAGATCAGCCTGCT−3′, R, 5′- GTGTATCTGGGAGCTGCGAG−3′, *fetal liver kinase 1* (*flk1*): F 5′- GGGTTTGGTTTTGGAAGGTT-3′, R, 5′- AGGAGCAAGCTGCATCATTT-3′, *gata4*: F 5′- CTGTCATCTCACTATGGGCA−3′, R, 5′- CCAAGTCCGAGCAGGAATTT−3′, *NK2 homeobox 5* (*nkx2-5*): F 5′- AAGCAACAGCGGTACCTGTC-3′, R, 5′- GCTGTCGCTTGCACTTGTAG−3′, *myosin heavy chain 7* (*myh7*): F 5′- TTGGCACGGACTGCGTCATC−3′, R, 5′- GAGCCTCCAGAGTTTGCTGAAGGA−3′, *glyceraldehyde-3-phosphate dehydrogenase* (*gapdh*): F 5′-CAGAAGACTGTGGATGGCCC-3′, R 5′-AGTGTAGC- CCAGGATGCCCT-3′.

### Immunofluorescence staining

Staining was performed as described (24). Primary antibodies: anti-tropomyosin (1:200, Sigma), anti-actinin (1:100, Abcam), anti-aurora B (1:200) (both BD Transduction Laboratories), rabbit polyclonal anti-troponin I, anti-cyclin A, anti-cyclin dependent kinase 1 (cdc2), anti-geminin (all 1:50, Santa Cruz Biotechnology), anti-phospho-histone H3 (Ser10) (1:200, Millipore), anti-pRb807/811 (1:100, Cell Signaling), anti-mAG (1:300, MBL), rat monoclonal anti-5-Bromo-2-deoxyuridine (BrdU) (1:100, Abcam). Immune complexes were detected with ALEXA 488- or ALEXA 594-conjugated secondary antibodies (1:200; Molecular Probes). DNA was visualized with DAPI'(4” 6'-diamidino-2-phenylindole, 0.5 μg/ml). For BrdU, cells were cultured in 30 μM BrdU (Sigma) (neonatal: last 24–48 h, adult: last 5 days).

### Culture and cardiac differentiation of human induced pluripotent stem cells (HiPSC)

The line hiPSC 19-9-11 (WISC Bank) was utilized to generate cardiomyocytes based on a previously published protocol (25). In brief, hiPSCs were maintained in mTeSR1 media (STEMCELL Technologies). When hiPSCs reached the proper confluency (~80%), they were passaged using trypsin-EDTA (Life Technologies) and plated at a density of 200,000 cells per well of a Matrigel (BD Biosciences)-coated 24-well plates containing mTeSR with 10 μM Rock Inhibitor (Y27632). After 24 h, media was changed with 0.5 mL mTeSR media without Rock inhibitor. At the following day, culture media was replaced with 50 nM XV (GSK3 inhibitor) in mTeSR media for hiPSC differentiation (Day 0). After 24 h, media was substituted with RPMI/B-27 minus insulin (Day 1). On Day 3, 50% of media was exchanged with RPMI/B-27 minus insulin and IWP-2 [Wnt signaling inhibitor, 7.5 μM (final concentration)]. On day 5, media was replenished with RPMI/B27 minus insulin. On day 7, media was changed with RPMI/B27. Then, media was renewed every 3 days (we kept the cells for 36 days). Next, cells were trypsinized with 0.25% (wt/vol) trypsin-EDTA and incubated the mixture in a 37°C, 5% CO_2_ incubator for 5 min. The cells were centrifuged at 200 x g for 5 min and the cell pellet was distributed in RPMI20 + 5 μM Y27632 on a laminin-coated coverslip (25 μg/mL). After 2 days, the cells were kept in RPMI/B-27 medium and maintained for the desired time (for 2 days). HiPSC-derived cardiomyocytes were then stimulated with small molecules to analyze their effect on cell cycle progression.

### MTS assay

Cell cytotoxicity was measured by an MTS assay (Abcam) according to the manufacture's instruction. Cardiomyocytes were treated with DMSO or individual small molecules for 72 h and absorbance was measured after MTS reagent addition at 4–0 - 500 nm using an Epoch microplate spectrophotometer (Biotek, USA).

### Cell count

In order to determine whether compounds increase the number of cells, 100,000 neonatal rat cardiomyocytes were seeded per well of 24-well plates. Cells were then cultured for 48 h in the presence of 20 μM ara C and 5% horse serum and subsequently treated with the indicated compounds in the presence of 0.2% FBS as indicated. The number of cells was determined 5 days post-treatment after trypsinization utilizing a TC20 cell counter (Bio-Rad, USA) according to the manufacturer's instructions.

### Statistical analysis

For immunofluorescence analyses, around 50 cardiomyocytes in five random fields of two different subpopulations were counted per experiment equaling a total cell number of around 500 cardiomyocytes. Data of at least three independent experiments are expressed as mean ± standard error of the mean (SEM). Results were analyzed by Graph Pad Prism (version 4.00, Graph Pad Software Inc.). Statistical significance was determined using a two-tailed Student's t-test or analysis of variance (ANOVA) were appropriate. The values of *p* < 0.05 were considered statistically significant.

## Results

### Live cell screening based on mouse stem cell-derived cardiomyocytes

**Figure 1:**
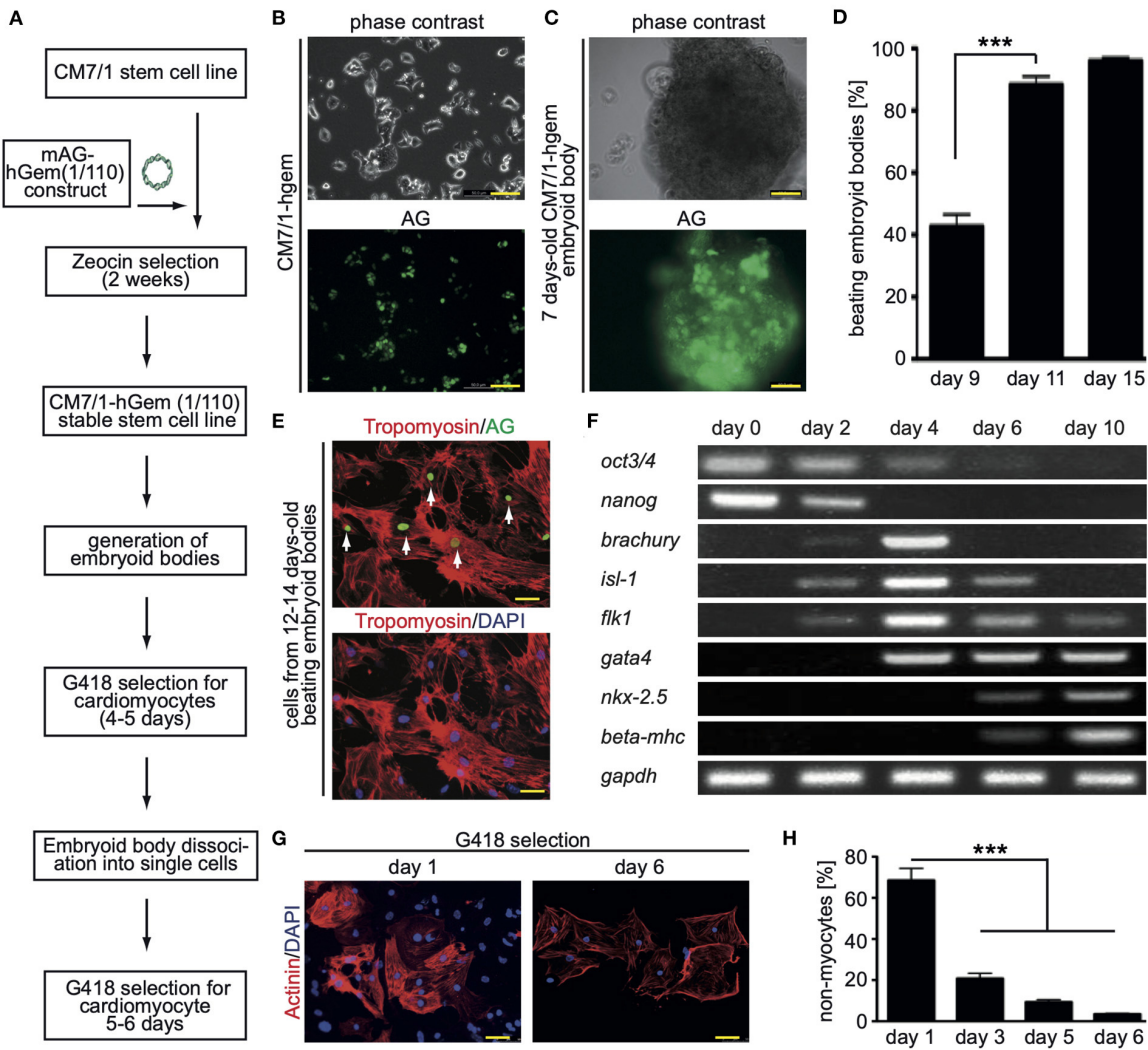
Generation of the CM7/1-hgem stem cell line and their differentiation. **(A)** Work flow of the generation of the CM7/1-hgem stem cell line. **(B)** Representative live pictures of nuclear expression of monomeric Azami Green (AG) in CM7/1 embryonic stem cells. **(C)** Representative live pictures of a 7-day-old differentiating embryonic body (EB) derived from CM7/1-hgem cells expressing AG. **(D)** Quantitative analysis of beating EBs at different time points (*n* = 3, mean ± SEM, ***: *p* < 0.001). **(E)** AG expression in CM7/1-hgem-derived cardiomyocytes. 12- to 14-day-old beating EBs were dissociated and stained for AG expression (green) and counterstained against cardiomyocyte-specific tropomyosin (red). DAPI was used to visualize nuclei (blue). **(F)** mRNA expression patterns of stem cell (oct3/4, nanog), mesodermal (brachyury), early progenitor (gata4), and cardiomyocyte (myh7) markers during differentiation of CM7/1-hgem stem cells into cardiomyocytes. **(G)** Enrichment for cardiomyocytes by treating 9-day-old EB with 400 μM G418 for 4 to 5 days. The EBs were then dissociated to single cell cultures and kept under 400 μM G418 for another 6 days. Representative examples of CM7/1-hgem cell-derived cardiomyocytes stained for sarcomeric alpha-actinin (red). Nuclei were visualized using DAPI (blue). **(H)** Quantitative analysis of **(G)** (*n* = 3, mean ± SEM, ***: *p* < 0.001). Scale bar = 50 μm in **(B,C,E,G)**.

In order to perform a screen for novel inducers of cardiomyocyte cell cycle activity, we have generated a mouse stem cell line that expresses a non-functional human geminin deletion mutant fused to a monomeric version of AG [mAG-hGem(1/110)] under the control of the ubiquitous CMV promoter (Figure 1A). Note, mAG-hGem(1/110) is only stable in S-/G2-/ and M-phase cells. In order to develop this cell line, we transfected the mouse ES cell line CM7/1 (26) with the plasmid mAG-hGem(1/110) (18), which allows selection for positive integration with Zeocin. The cell line CM7/1 expresses the neomycin-phospho-transferase gene under the control of the cardiomyocyte-specific α*-myosin heavy chain* promoter. This allows to obtain stem cell-derived cardiomyocyte cultures with a purity of > 99% after G418-treatment (26). This approach resulted in the establishment of the cell line CM7/1-hgem expressing AG (Figure 1B), which can be induced to differentiate into well beating EBs (Figures 1C,D). To ensure that cardiomyocytes derived from CM7/1-hgem express mAG-hGem(1/110), 12–14 days-old beating EBs were dissociated and seeded cells were stained for AG and a cardiomyocyte-specific marker (Figure 1E). This analysis revealed that 22.3 ± 6.8% of cardiomyocytes expressed mAG-hGem(1/110). That mAG-hGem(1/110) expression does not interfere with cardiogenesis of CM7/1-hgem was validated by normal temporal mRNA expression patterns of stem cell (*oct3/4, nanog*), mesodermal (*brachyury*), early progenitor (*gata4*), and cardiomyocyte (*myh7*) markers (Figure 1F). The actual screen for novel inducers of cardiomyocyte cell cycle activity was performed with cardiomyocytes obtained by treating CM7/1-hgem EBs at day 9 of differentiation with G418, dissociation of these EBs at day 12 to 14, plating the single cells on fibronectin-coated plates, and culturing them for another 6 days in the presence of G418. Immunofluorescence analysis revealed that the percentage of non-myocytes in the single cell culture decreased from 68.6 ± 5.8% at day 1 to < 3.5 ± 0.4% at day 6 (mean ± SEM, ^***^: *p* < 0.001, Figures 1G,H).

**Figure 2:**
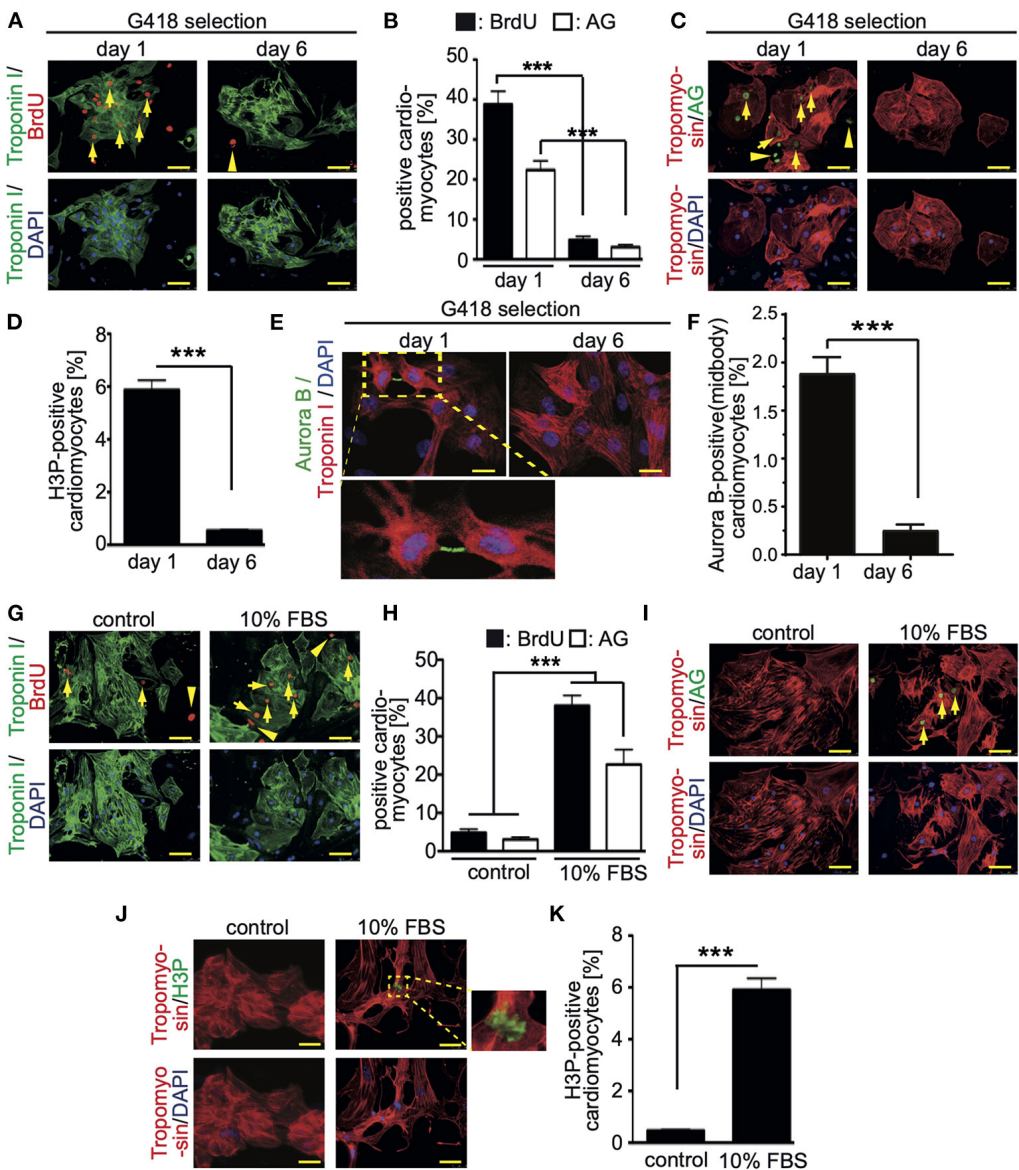
Cell cycle activity in CM7/1-hgem derived cardiomyocytes. **(A,C)** The number of BrdU- and Azami Green (AG)-positive CM7/1-hgem-derived cardiomyocytes decreased during differentiation. Representative examples of CM7/1-hgem-derived cardiomyocytes stained for troponin I (green) or tropomyosin (red) (cardiomyocyte-specific), BrdU (red) **(A)** or AG (green) **(C)**, and DAPI (nuclei, blue). Scale bar: 50 μm. *n* = 6. **(B)** Quantitative analysis of **(A,C)** (*n* = 6, mean ± SEM, ***:*p* < 0.001). **(D)** Quantitative analysis of H3P-positive CM7/1-hgem-derived cardiomyocytes (*n* = 6, mean ± SEM, ***:*p* < 0.001). **(E)** Representative examples of CM7/1-hgem-derived cardiomyocytes stained for troponin I (red) (cardiomyocyte-specific), Aurora B (green), and DAPI (nuclei, blue). Scale bar: 20 μm. **(F)** Quantitative analysis of Aurora B-positive CM7/1-hgem-derived cardiomyocytes at the midbody (*n* = 6, mean ± SEM, ***:*p* < 0.001). **(G–I)** Stimulation of cell cycle progression in CM7/1-hgem-derived cardiomyocytes by 10% FBS. Representative examples of CM7/1-hgem-derived cardiomyocytes stained for troponin I (green) **(G)** or tropomyosin (red) **(I)** (cardiomyocyte-specific), BrdU (red) **(G)** or AG (green) **(I)**, and DAPI (nuclei, blue). Scale bar: 50 μm. *n* = 3. **(F)** Quantitative analysis of **(G,I)** (*n* = 6, mean ± SEM, ***:*p* < 0.001). **(J)** Representative examples of CM7/1-hgem-derived cardiomyocytes stained for tropomyosin (red) (cardiomyocyte-specific), H3P (green), and DAPI (nuclei, blue). Scale bar: 50 μm. *n* = 6. **(K)** Quantitative analysis of **(J)** (*n* = 6, mean ± SEM, ***: *p* < 0.001).

To assess the base level of CM7/1-hgem-derived cardiomyocyte cell cycle activity, BrdU incorporation (24 h) assays were performed and the number of mAG-hGem(1/110)-, H3P (histone H3 phosphorylation on serine 10)-positive as well as Aurora B-positive cardiomyocytes was determined at day 1 (non-selected) and day 6 (selected) post-dissociation. The number of cardiomyocytes incorporating BrdU decreased from 38.8 ± 3.5% at day 1 to 4.8 ± 0.92% at day 6 (mean ± SEM, *p* < 0.01, Figures 2A,B). Assuming that completion of one cell cycle lasts less than 24 hours, all cycling cardiomyocytes should be BrdU-positive. The percentage of cardiomyocytes expressing mAG-hGem(1/110) decreased from 22.3 ± 3% to 3 ± 0.6% (mean ± SEM, *p* < 0.01, Figures 2B,C). The overall number of mAG-hGem(1/110)-positive cells is as expected lower than BrdU-positive cells, as mAG-hGem(1/110) is only stable in S-/G2-/ and M-phase cells. The number of H3P-positive cardiomyocytes decreased from 5.9 ± 0.45% to 0.45 ± 0.1% (Figure 2D). Note, histone H3 is only phosphorylated in late G2-phase cells and M-phase cells until late anaphase. Finally, the number of Aurora B-positive cardiomyocytes at the midbody decreased from 1.89 ± 0.1% to 0.25 ± 0.03% (Figures 2E,F). Note, Aurora B is only a marker for cytokinesis when localized to the midbody or cleavage furrow. The overall decrease of cycling cardiomyocytes from day 1 to day 6 is in agreement with the general observation that the proliferation rate of ES cell-derived cardiomyocytes decreases over time in culture (27).

In order to test whether cell cycle activity in CM7/1-hgem-derived cardiomyocytes can be promoted, cells were stimulated for 3 days with 10% FBS, which is known to promote cell cycle activity in fetal, neonatal, and adult mammalian cardiomyocytes (24, 28). FBS treatment induced in 37 ± 3.8% of cardiomyocytes BrdU incorporation (Figures 2G,H), in 21 ± 4.4% mAG-hGem(1/110) expression (Figures 2H,I), and in 6 ± 0.8% histone H3 phosphorylation (Figures 2J,K) compared to 4.8 ± 0.9% (BrdU), 3 ± 0.6% (AG), and 0.49 ± 0.09% (H3P) upon DMSO treatment, respectively (mean ± SEM, *p* < 0.01, Figures 2H,K).

### Screening for small molecules inducing CM7/1-hgem-derived cardiomyocyte cell cycle activity

**Figure 3:**
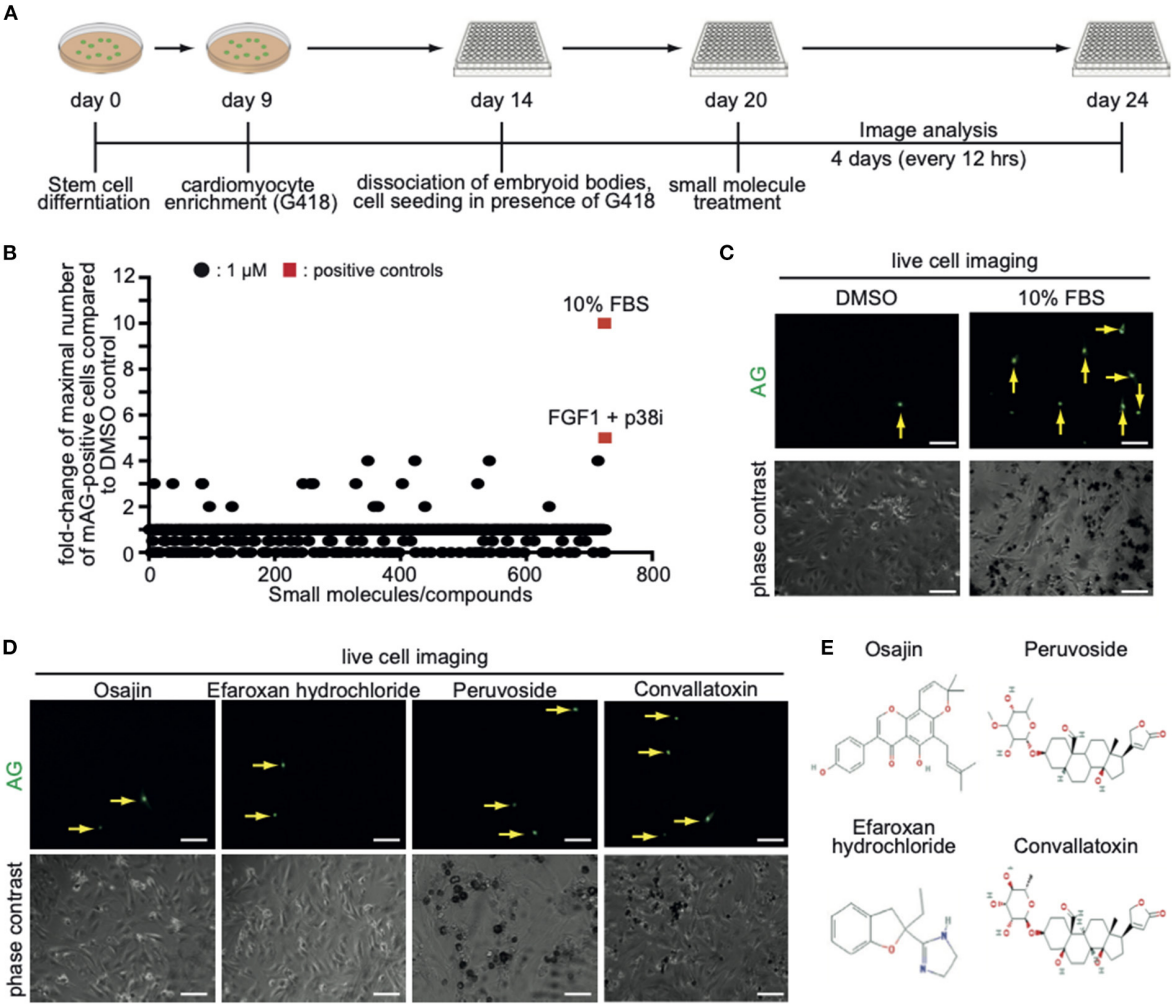
Screening of a small molecule library identifies novel compounds promoting CM7/1-hgem-derived cardiomyocyte cell cycle progression. **(A)** Workflow of the applied screening strategy. **(B)** Quantitative analysis of Azami Green (AG) expression in the screen of a Microsource spectrum small molecule screening library. **(C)** Representative live pictures of AG expression in control or 10% FBS-treated CM7/1-hgem-derived cardiomyocytes. Scale bar: 100 μm. **(D)** Representative live pictures of AG expression in osajin-, efaroxan hydrochloride-, peruvoside-, and convallatoxin-treated CM7/1-hgem-derived cardiomyocytes. Scale bar: 100 μm**. (E)** Molecular design of small molecule hits.

In order to identify novel inducers of cardiomyocyte cell cycle activity, a subset of compounds of the small molecule library “Spectrum Collection” from MicroSource Discovery Systems, Inc. (Gaylordsville, CT) was utilized. This library presents 2,560 compounds and includes drugs from three sources: (1) US drug collection of 1,040 drugs that have reached clinical trial stages in the USA whereby each compound has been assigned USAN or USP status. (2) An International Drug Collection of 240 drugs that are marketed in Europe and/or Asia. (3) The rest is a unique collection of pure natural products and their derivatives. Natural Products include simple and complex oxygen heterocycles, alkaloids, sesquiterpenes, diterpenes, pentacyclic triterpenes, sterols, and many other diverse representatives. For each compound, data can be obtained regarding its structure, CAS #, formula, molecular weight, biological profile, as well as generic and market name. In addition, literature references are available describing the use and toxicology of the individual compounds. CM7/1-hgem-derived EB's were dissociated on day 14, seeded at 15,000 cells per well in a 96-well plate (flat glass-bottomed), and cultured for 6 days in the presence of G418. Subsequently, cells were treated with a subset of compounds of the small molecule library “Spectrum Collection” at a concentration of 1 μM. DMSO served as a negative control, 10% FBS and FGF1/p38i as positive controls. mAG-hGem(1/110)-positive cells per field were recorded for 4 days at intervals of 12 h (Figure 3A). As this was a pilot study, recording (image acquisition) and image analysis were performed manually, and the screen was performed only once. Note, to minimize the manual labor, only around 100 cells in the center of each well were evaluated utilizing a 20x objective. Data are represented as a fold-change increase of the observed maximal number of mAG-hGem(1/110)-positive cells per field in comparison to the maximal number in DMSO-treated cultures (Figure 3B). Note, in cases when compound treatment resulted in no mAG-hGem(1/110)-positive cell at markedly more time points (≥ 4) than in the DMSO control (1 of 8), the fold-change was defined as 0.5-fold. The number of observed mAG-hGem(1/110)-positive cells at the different 12 h time points for each treatment are provided in Supplementary Table 1. Our analysis revealed that 10% FBS as well as FGF1/p38i efficiently induced mAG-hGem(1/110) expression in CM7/1-hgem-derived cells enriched for cardiomyocytes (~10 fold and ~ 5-fold, respectively, Figures 3B,C). While most of the 722 tested compounds had no positive effect on mAG-hGem(1/110) expression, 19 compounds induced mAG-hGem(1/110) expression in at least twice as many cells as upon DMSO treatment (Figure 3B, Supplementary Table 1). The most effective compounds were osajin, efaroxan hydrochloride, peruvoside, and convallatoxin (all 4-fold) (Figures 3D,E).

### Cell cycle-promoting effect of identified compounds on neonatal and adult primary rat cardiomyocytes

Stem cell-derived cardiomyocytes are considered immature exhibiting a behavior similar to late fetal cardiomyocytes (29). In order to assess whether the here identified 19 compounds could promote cell cycle progression also in more mature cardiomyocytes, neonatal rat cardiomyocytes were stimulated and analyzed 3 days later for BrdU incorporation (BrdU pulse-labeled for the final 24 h). For this purpose, cardiomyocytes were isolated from postnatal day 3 (P3) rats and stimulated once with the individual small molecules at concentrations of 50 nM, 250 nM, 1 μM, and 5 μM (Supplementary Figure 1).

**Figure 4:**
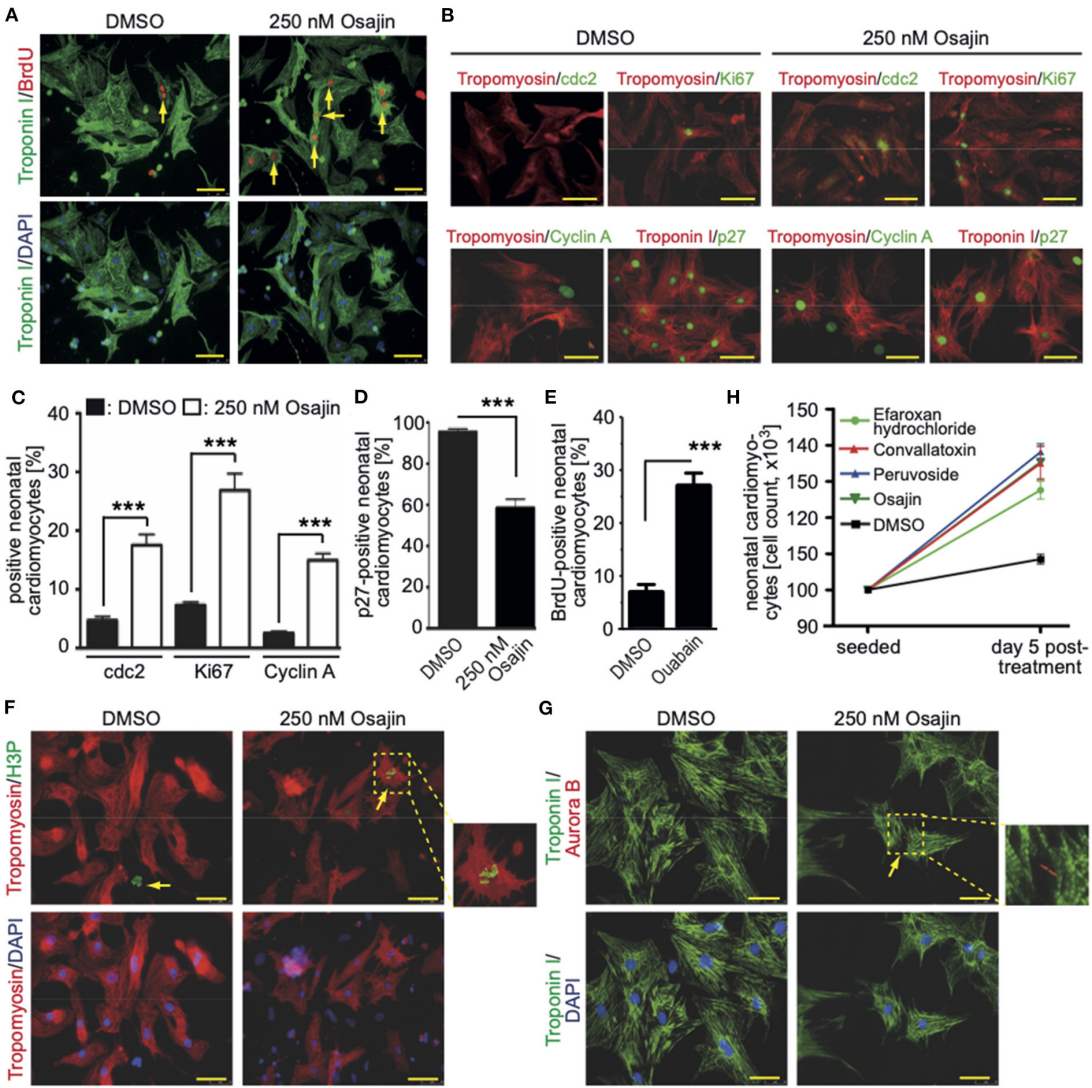
Promoting neonatal cardiomyocyte cell cycle progression by small molecules (Positive hits). **(A)** Osajin induced BrdU incorporation of P3 neonatal cardiomyocytes (*n* = 6). Representative example of neonatal cardiomyocytes stained for BrdU (red) and troponin I (green, cardiomyocyte-specific). Examples are indicated by arrows. DNA was visualized using DAPI (blue). **(B)** Osajin treatment induced increased expression of cell cycle promoting factors and decreased expression of cell cycle inhibitors (immunofluorescence analyses at 48 h after stimulation). Representative example of neonatal cardiomyocytes stained for cdc2, Ki67, cyclin A, or p27 (green) and tropomyosin (red, cardiomyocyte-specific). DNA was visualized using DAPI (blue). **(C,D)** Quantitative analysis of **(B)** (*n* = 6, mean ± SEM, ***: *p* < 0.001). **(E)** Quantitative analysis of the number of BrdU-positive cardiomyocytes after ouabain treatment (*n* = 6, mean ± SEM, ***: *p* < 0.001). **(F,G)** Representative examples of osajin-stimulated neonatal cardiomyocytes stained for tropomyosin (red) or troponin I (green, both cardiomyocyte-specific) undergoing mitosis (H3P, green) and cytokinesis (Aurora B, red). Examples are indicated by arrows. Arrowhead: H3P-positive non-myocyte. DNA was visualized using DAPI (blue). **(H)** Quantitative analysis of the increase in cell number five days after treatment with the indicated compounds (*n* = 5, mean ± SD). Scale bars: 50 μm.

Subsequently, cardiomyocytes were treated with the optimal concentrations of the individual compounds, cultured for 3 days and pulse-labeled with BrdU for the final 48 hours to assess also cardiomyocytes that already entered S phase after 24 h to 48 h post-treatment. In Supplementary Figure 1 (data from six independent experiments per parameter) the data for the optimal concentration of each of the 19 compounds is provided whereby treatment with 250 nM osajin was most efficient in inducing BrdU incorporation in P3 rat cardiomyocytes (30.3 ± 2.4% *vs*. DMSO: 10.5 ± 0.9%, *p* < 0.01, Figure 4A). A deeper analysis showed that osajin treatment induced the expression of cell cycle perpetuating factors like cdc2, cyclin A, and Ki67 (Figures 4B,C) and the downregulation of the cell cycle inhibitor p27 (Figures 4B,D). Notably, also the cardiac glycosides convallotoxin and peruvoside, efficiently induced BrdU incorporation (Supplementary Figure 1). Thus, we also tested the effect of the cardiac glycoside ouabain on P3 cardiomyocyte proliferation. Similar to the other cardiac glycosides, ouabain induced efficiently BrdU incorporation (28.8 ± 2.9% vs. DMSO: 9.1 ± 0.7%, *p* < 0.01, Figure 4E). Analyses of H3P revealed that all tested cardiac glycosides and all other compounds stimulating BrdU incorporation, except merogedunin, also induced cell cycle progression into G2/M-phase (Table 1). For example, treatment with 250 nM osajin induced the number of H3P-positive cardiomyocytes ~5-fold compared to DMSO (1.86 ± 0.3% *vs*. DMSO: 0.37 ± 0.1%, *p* < 0.01) after 3 days of stimulation (Figure 4F). The analysis of aurora B expression suggests that most compounds, including osajin (1.73 ± 0.3%), efaroxan hydrochloride (1.65 ± 0.3%) as well as the cardiac glycosides peruvoside (1.36 ± 0.2%) and convallotoxin (1.28 ± 0.2% *vs*. DMSO: 0.35 ± 0.1%, *p* < 0.01), also induce cytokinesis (Table 1). Notably, cardiomyocytes exhibited in most cases a two-sided cleavage furrow, as shown for osajin (Figure 4G). Finally, cell count experiments revealed that the treatment of all compounds resulted 5 days post-treatment in a significantly increased cell number (Figure 4H). Notably, none of these compounds exhibited cytotoxic effects on cardiomyocytes based on MTS assays (Supplementary Figure 2). Taken together, our data suggest that our screening system allows the identification of molecules with the potential to promote neonatal cardiomyocyte proliferation such as cardiac glycosides.

**Table 1:** Cell cycle parameters in neonatal rat cardiomyocytes treated with selected small molecules.

**Small molecule**	**conc**.	**48 h BrdU [%]**	**H3P [%]**	**aurora B [%]**
Osajin	0.25 μM	30.3 ± 2.4	1.86 ± 0.3	1.73 ± 0.3
Efaroxan Hydrochloride	1 μM	29.6 ± 2.1	1.76 ± 0.3	1.65 ± 0.3
Peruvoside	1 μM	30.0 ± 2.6	1.32 ± 0.2	1.36 ± 0.2
Convallatoxin	1 μM	29.3 ± 3.0	1.35 ± 0.3	1.28 ± 0.2
Valeryl salycilate	0.25 μM	27.0 ± 2.5	1.35 ± 0.1	1.21 ± 0.1
Idebenone	1 μM	22.0 ± 2.6	0.97 ± 0.1	0.92 ± 0.2
Lobaric acid	5 μM	22.0 ± 1.5	0.92 ± 0.2	0.85 ± 0.2
PIDOTIMOD	1 μM	21.3 ± 2.0	0.85 ± 0.1	0.75 ± 0.1
Ferulic acid	1 μM	22.6 ± 2.0	0.80 ± 0.2	0.75 ± 0.2
Quircitrin	1 μM	22.6 ± 0.8	0.75 ± 0.2	0.75 ± 0.1
Glutathione	1 μM	24.6 ± 2.3	0.60 ± 0.2	0.65 ± 0.2
Acedoben	1 μM	20.0 ± 3.2	0.65 ± 0.1	0.61 ± 0.1
Isaxonine	1 μM	21.6 ± 2.4	0.60 ± 0.2	0.50 ± 0.1
Protoporphyrin	1 μM	16.6 ± 3.8	0.55 ± 0.1	0.50 ± 0.1
N-methylanthranilic acid	1 μM	17.3 ± 1.8	0.60 ± 0.1	0.50 ± 0.1
Gitoxin	1 μM	19.0 ± 2.1	0.60 ± 0.1	0.50 ± 0.1
Tiratricol	1 μM	25.3 ± 3.7	0.55 ± 0.1	0.47 ± 0.1
Bilirubin	1 μM	18.6 ± 1.9	0.56 ± 0.2	0.45 ± 0.1
Merogedunin	1 μM	19.0 ± 1.2	0.35 ± 0.1	0.30 ± 0.1
DMSO		10.5 ± 0.9	0.37 ± 0.1	0.35 ± 0.1

**Figure 5:**
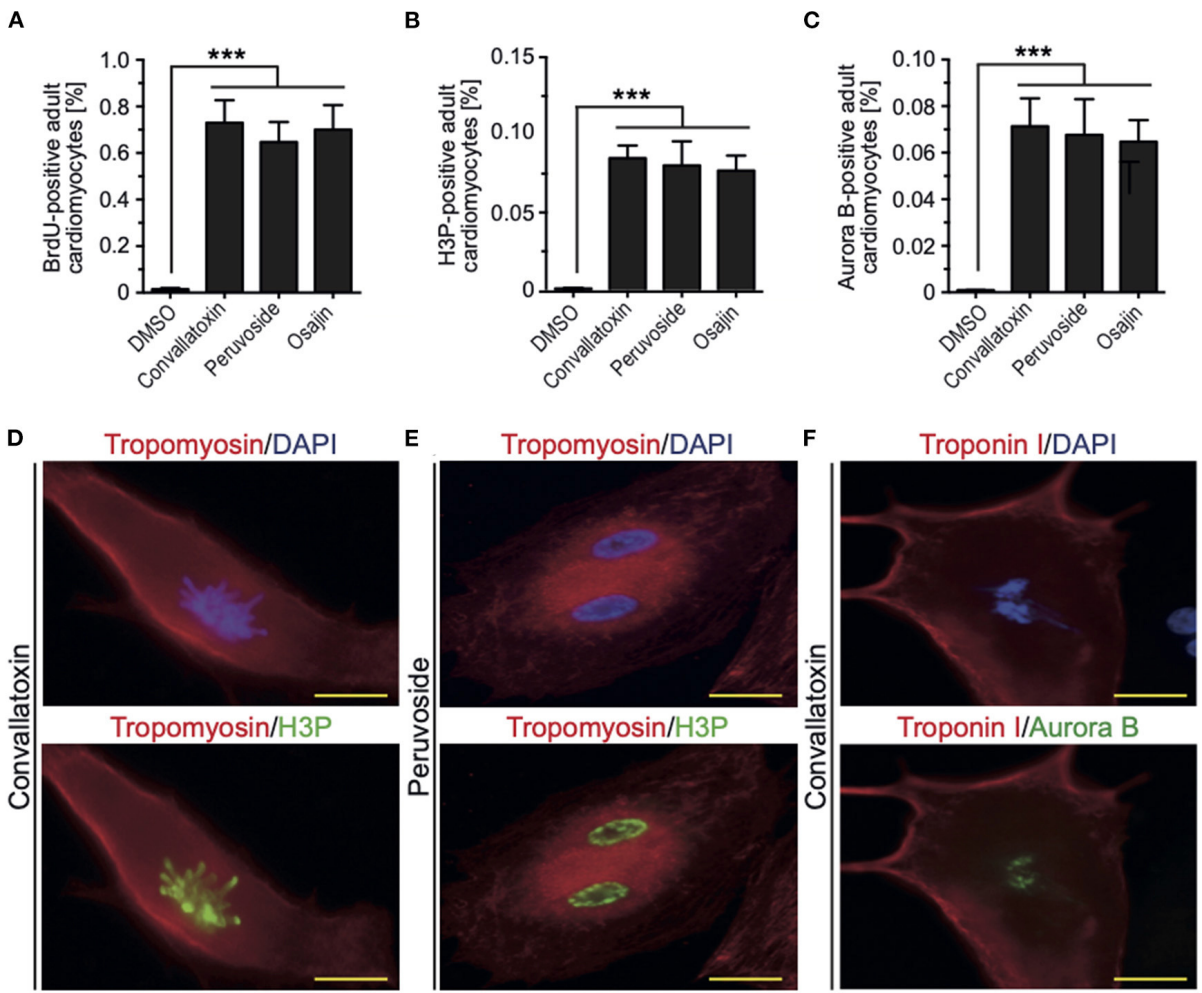
Osajin and cardiac glycosides promote adult cardiomyocyte cell cycle progression. **(A–C)** Quantitative analysis of cell cycle progression [BrdU incorporation, **(A)**], mitosis [H3P, **(B)**], and cytokinesis [Aurora B, **(C)**] (*n* = 6, mean ± SEM, ***: *p* < 0.001). **(D–F)** Representative examples of adult rat cardiomyocytes (tropomyosin- or troponin I-positive) utilized for the quantitative analysis in **(B,C)**. **(D)** Mononucleated adult rat cardiomyocyte stained for H3P in pro-metaphase. **(E)** Binucleated adult rat cardiomyocyte stained for H3P in G2 to prophase. **(F)** Binucleated adult rat cardiomyocyte stained for aurora B in G2 to prophase.

To determine if cardiac glycosides can also promote cell cycle progression in adult rat cardiomyocytes, ventricular cardiomyocytes from 12-week-old rats were stimulated and analyzed for BrdU incorporation, histone H3 phosphorylation, and aurora B expression. Osajin as well as the cardiac glycosides peruvoside and convallotoxin efficiently induced cell cycle re-entry in adult rat cardiomyocytes (all > 0.6% BrdU-, > 0.07% H3P-, and > 0.06% aurora B-positive cardiomyocytes compared to < 0.002% for all parameters upon DMSO treatment, Figures 5A–C). Notably, cell cycle activity was observed in mono- as well as binucleated adult rat cardiomyocytes (Figures 5D–F). Collectively, these data show that cardiac glycosides can promote adult cardiomyocyte cell cycle progression.

### Identified compounds promote cell cycle progression of HiPSC-derived cardiomyocytes

**Figure 6:**
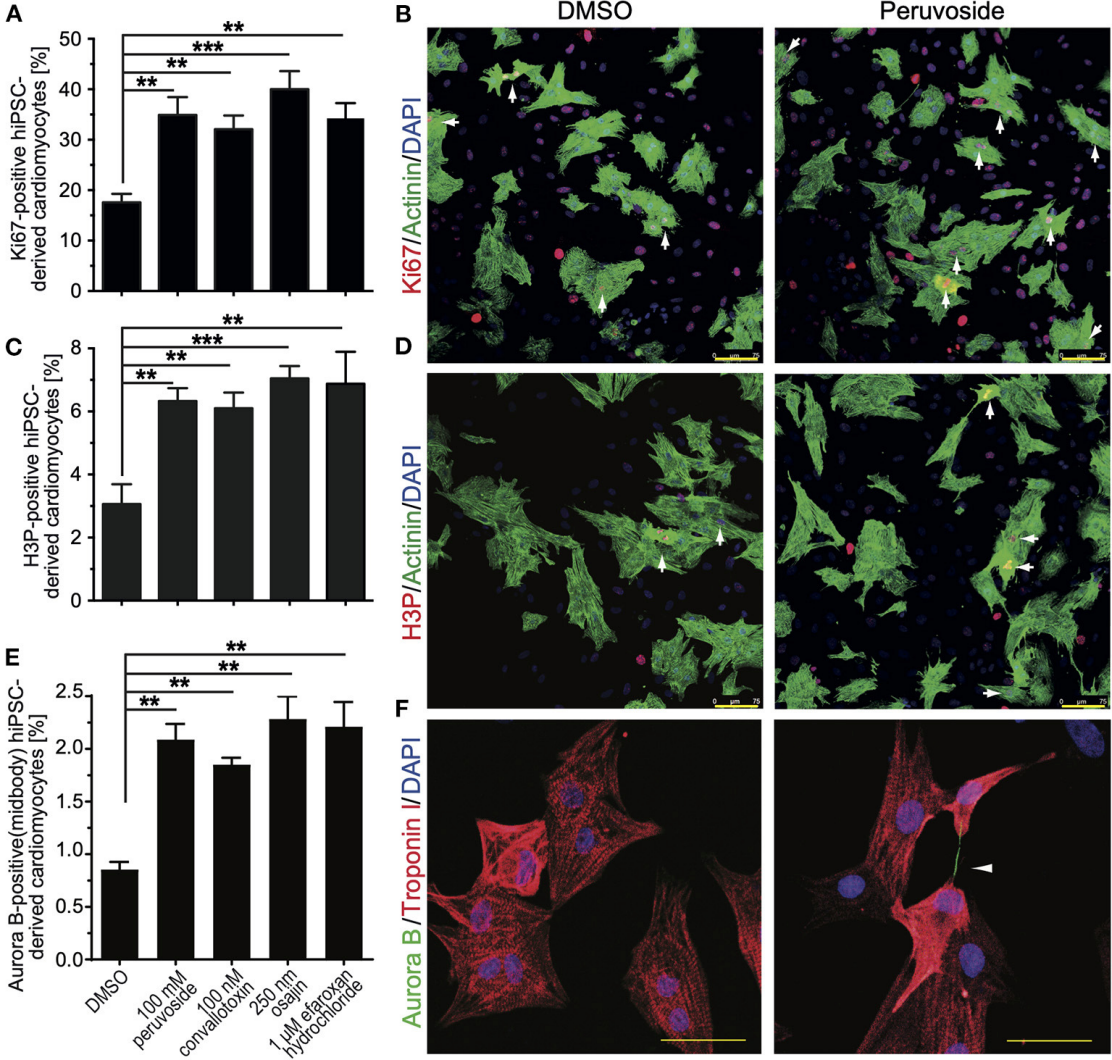
Small molecules (positive hits) promote cell cycle progression in hiPSC-derived cardiomyocytes. HiPSC-derived cardiomyocytes were stimulated with the indicated compounds for 2 days and subsequently analyzed in regards to cell cycle activity. **(A)** Quantitative analysis of the percentage of Ki67-positive cardiomyocytes (*n* = 6, mean ± SEM, **: *p* < 0.01, ***: *p* < 0.001). **(B)** Representative examples were utilized for the analysis in **(A)**. Ki67: red; Actinin: green (cardiomyocyte-specific). DNA (DAPI): blue. Examples of Ki67-positive cardiomyocytes are indicated by arrows. **(C)** Quantitative analysis of the percentage of H3P-positive cardiomyocytes (*n* = 6, mean ± SEM, **: *p* < 0.01, ***: *p* < 0.001). **(D)** Representative examples were utilized for the analysis in **(C)**. H3P: red; Actinin: green (cardiomyocyte-specific). DNA (DAPI): blue. Examples of H3P-positive cardiomyocytes are indicated by arrows. Scale bars: 75 μm. **(E)** Quantitative analysis of the percentage of Aurora B-positive cardiomyocytes at the midbody (*n* = 6, mean ± SEM, **: *p* < 0.01, ***: *p* < 0.001). **(F)** Representative examples were utilized for the analysis in **(E)**. Aurora B: Green; Troponin I: green (cardiomyocyte-specific). DNA (DAPI): blue. Example of an Aurora B-positive cardiomyocyte at the midbody is indicated by an arrowhead. Scale bars: 50 μm.

Aiming at evaluating the translation potential of the identified compounds, the effect of the positive hits, including cardiac glycosides, on cell cycle progression in hiPSC-derived cardiomyocytes was assessed. HiPSC-derived cardiomyocytes were treated with DMSO or peruvoside (100 nM), convallotoxin (100 nM), osajin (250 nM), and efaroxan hydrochloride (1 μM) and analyzed for changes in the expression of Ki67 or phosphorylation of histone H3 (H3P) 48 h post-treatment (Figures 6A–D). Pervuvoside significantly increased the number of both Ki67- and H3P-positive cardiomyocytes compared to DMSO (Ki67: 34.90 ± 2% *vs*. DMSO: 17.57 ± 0.97%, *p* < 0.01; H3P: 6.3 ± 0.23% *vs*. DMSO: 3.06 ± 0.36%, *p* < 0.01). Similarly, all other tested compounds exhibited a positive effect on cell cycle progression in hiPSC-derived cardiomyocytes (convallotoxin: Ki67: 32.07 ± 1.57%, H3P: 6.1 ± 0.28%; osajin: Ki67: 40.00 ± 2.0%, H3P: 7.04 ± 0.22%; efaroxan hydrochloride: Ki67: 34.25 ± 1.7%, H3P: 6.88 ± 0.58%; all *p* < 0.01, Figures 6A–D). Finally, we analyzed Aurora B-positive cardiomyocytes at midbody indicative for cell division. We found that pervuvoside significantly increased cardiomyocytes in cytokinesis compared to DMSO (Aurora B at midbody: 2.08 ± 0.08% *vs*. DMSO: 0.85 ± 0.04%, *p* < 0.01) (Figures 6E,F). Similarly, all other tested compounds exhibited a positive effect on cardiomyocyte cytokinesis (convallotoxin: 1.85 ±0.04%; osajin: 2.28 ± 0.12%; efaroxan hydrochloride: 2.21 ± 0.13%; all *p* < 0.01, Figure 6E). Taken together, these data suggest that cardiac glycosides, as well as osajin and efaroxan hydrochloride, also positively affect cell cycle progression in hiPSC-derived cardiomyocytes.

### Inhibition of PTEN and GSK-3β enhance cell cycle progression induced by cardiac glycosides and osajin

**Figure 7:**
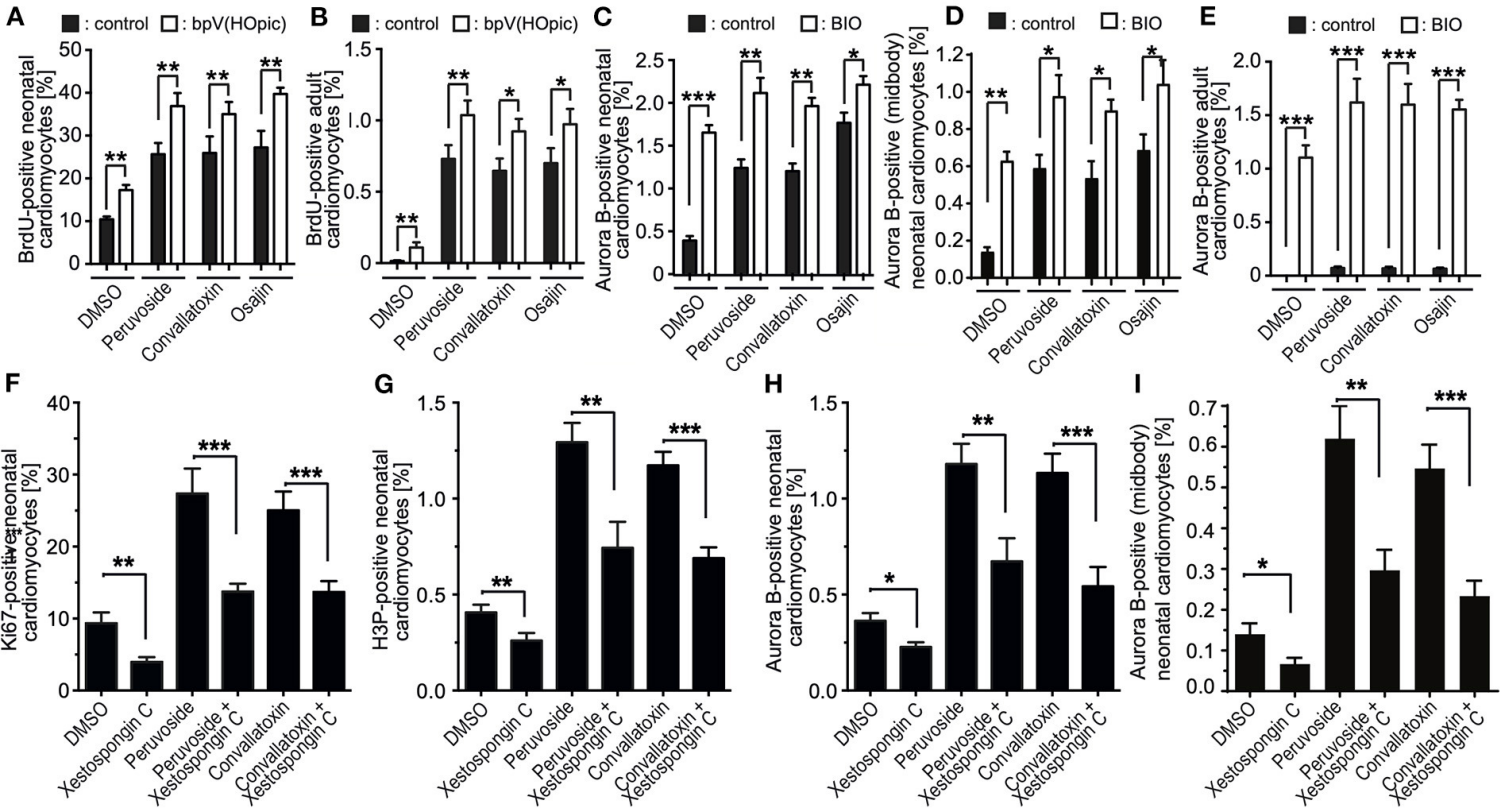
Mechanistic analysis of compound-enhanced cell cycle progression. **(A–E)** Inhibition of PTEN, as well as GSK-3β enhances cell cycle progression induced by cardiac glycosides and osajin. Quantitative analysis of BrdU- **(A,B)** and Aurora B- **(C,E)**, Aurora B at the midbody in neonatal cardiomyocytes **(D)**, positive neonatal **(A,C)** and adult **(B,E)** cardiomyocytes upon stimulation with cardiac glycosides and osajin in the presence and absence of PTEN inhibitor bpV (HOpic) **(A,B)** or GSK3-β inhibitor BIO **(C–E)**. *n* = 6, mean ± SEM, *: *p* < 0.1, **: *p* < 0.01, ***: *p* < 0.001. **(F–I)** Inhibition of IP3 receptor impedes peruvoside- and convallotoxin-induced cardiomyocyte cell cycle progression. Neonatal rat cardiomyocytes were stimulated with glycosides in the absence or presence of IP3 receptor inhibitor xestospongin C (1 μM) and the number of Ki67-, H3P-, and aurora B-positive cardiomyocytes were evaluated. Quantitative analysis of the percentage of Ki67- [cell cycle progression, **(F)**], H3P- [mitosis, **(G)**], and aurora B- [cytokinesis, **(H,I)**] positive cardiomyocytes (*n* = 6, mean ± SEM, *: *p* < 0.05), **: *p* < 0.01, ***: *p* < 0.001.

Previously, others and we have shown that the phosphoinositide 3-kinase (PI3K)-Akt pathway is implicated in the regulation of cardiomyocyte proliferation (14, 24, 30) and phosphatase and tensin homolog (PTEN), an inhibitor of the PI3K pathway, inhibits periostin-induced cardiomyocyte cell cycle activity (30). Recently, it was shown that loss of PTEN promotes cardiomyocyte proliferation and cardiac repair after MI, suggesting the pathway has a substantial role in cardiomyocyte proliferation (31). Thus, we have tested whether bpv(HOpic), a potent inhibitor of PTEN, enhances the effect of osajin and the cardiac glycosides on BrdU incorporation in neonatal and adult cardiomyocytes. As expected, bpv(HOpic) increased the number of BrdU-positive cardiomyocytes in the control, but was less efficient than osajin and the cardiac glucosides (Figures 7A,B). Notably, bpv(HOpic) significantly enhanced the effect of osajin and the cardiac glucosides on inducing BrdU incorporation in primary neonatal (Figure 7A) as well as adult (Figure 7B) rat cardiomyocytes. In addition, we have previously demonstrated that the small molecule BIO, a specific inhibitor of glycogen synthase kinase-3 (GSK-3), promotes cell cycle progression into mitosis in neonatal and adult mammalian cardiomyocytes (14, 32). Thus, we have tested whether BIO enhances the effect of osajin and the cardiac glycosides in regards to aurora B expression in neonatal and adult cardiomyocytes (Figures 7C,E). While BIO had a moderate effect on aurora B expression in neonatal cardiomyocytes (Figure 7C) and aurora B-positive neonatal cardiomyocytes at the midbody (Figure 7D), it markedly enhanced aurora B expression in adult cardiomyocytes stimulated with osajin or the cardiac glycosides (Figure 7E). Yet, no adult cardiomyocytes positive for Aurora B at the midbody or cleavage furrow was observed. Yet, considering the rare observation of aurora B-positive adult cardiomyocytes and the short existence of the midbody, it is also rather unlikely to find an adult cardiomyocyte in this stage.

### IP3 receptor inhibition reduces the cell cycle-promoting effect of cardiac glycosides

Cardiac glycosides increase the output force of the heart and decrease its rate of contractions by acting on the cellular sodium-potassium ATPase pump, Na^+^K^+^ATPase (20). As Na^+^K^+^ATPase is known to specifically regulate calcium transients *via* the inositol trisphosphate (IP3) receptors (33–35), we have tested the effect of the selective IP3 receptor antagonist xestospongin C (36) on cardiac glycoside-enhanced cardiomyocyte cell cycle progression. For this purpose, P3 rat cardiomyocytes were stimulated with glycosides in the absence or presence of 1 μM xestospongin C and the number of Ki67-, H3P-, aurora B-positive cardiomyocytes as well as cardiomyocytes positive for aurora B at the midbody were evaluated. This analysis revealed that the presence of xestospongin C significantly reduced the positive effect of peruvoside and convallotoxin on neonatal cardiomyocyte cell cycle progression (Figures 7F–I). These data suggest that Ca2+ levels regulated by glycosides might be important for the cell cycle promoting effect of cardiac glycosides.

## Discussion

Here we developed a fluorescence-based live imaging screening assay based on mouse stem cells and the Fucci system to identify new inducers of cardiomyocyte proliferation. This system eliminates the need for immunofluorescence staining, incorporation of nucleotide analogs or cell count assays and allows to capture events that may develop at different times post-treatment, which may be potentially overlooked by end-point assays. As the system utilizes a ubiquitous promoter, this system can also be utilized to identify new inducers of proliferation of other cell types, such as neurons. Validation of positive hits utilizing several independent assays in primary neonatal and adult mammalian cardiomyocytes identified among others cardiac glycosides as a novel potential inducer of mammalian postnatal cardiomyocytes. In addition, the effect of cardiac glycosides on cardiomyocyte cell cycle progression was enhanced by inhibition of PTEN as well as GSK-3β and inhibited by IP3 receptor antagonist xestospongin C, which is known to inhibit IP3-mediated Ca^2+^ release from endo- and sarcoplasmic reticulum (36).

The here developed assay proved to be valid to identify novel compounds to enhance cardiomyocyte cell cycle progression. While our study is a pilot study being limited in a number of compounds, a number of analyzed cells per time point, and repetitions, pipette robots and automated image analysis solutions will allow in the future unbiased, in-depth analyses of high-content compound libraries.

To date, no direct data are available regarding the effect of cardiac glycoside on cardiomyocyte proliferation. In general, cardiac glycosides inhibit the sodium-potassium pump resulting in an increased calcium concentration inside the cell. It is well known that Na^+^K^+^ATPase specifically regulates calcium transients *via* the IP3 receptors (33–35) and our data show that inhibition of the IP3 receptors with xestospongin C inhibits the cell cycle-promoting effect of glycosides. Notably, calcium plays, in general, a crucial role in cell proliferation and aberrant Ca^2+^-signaling and loss of intracellular Ca^2+^ homeostasis contributes to tumor initiation and proliferation (37, 38). While cardiac glycosides became recently popular to inhibit tumor growth (39), there is evidence that cardiac glycosides can promote cell proliferation at lower, subsaturating concentrations. It has been reported that low concentrations of cardiac glycosides stimulate cell proliferation in astrocytes (40), vascular smooth muscle cells (41), renal tubule cells (42–44), Sertoli cells (45), human endothelial cells (46), and human umbilical vein smooth muscle cells (47). In addition, it has been indicated that voltage-gated L-type Ca^2+^ channels blockers enhance hiPSC-derived cardiomyocyte proliferation (48). Further, it has been reported that modulation of calcium channel activity controls proliferation vs. differentiation of cardiac progenitor cells (49). Finally, it has been suggested that cardiac glycosides can interfere with nuclear receptor signaling (19). For example, it has been shown that the cardiac glycosides digoxin and lanatoside C induce both the expression of peroxisome proliferator-activated receptor (PPAR) δ (50–52), which previously has been shown to promote cardiomyocyte proliferation (14). Taken together, our data indicate that cardiac glycosides can enhance cell cycle progression in cardiomyocytes.

Besides cardiac glycosides, we have identified the flavonoid osajin as a potent inducer of postnatal cardiomyocyte cell cycle progression. Previously, it has been shown that osajin exhibits a potential cardioprotective role in ischemia-reperfusion-induced injury in rat hearts. This cardioprotective role has been attributed to the suppression of oxidative stress resulting in improved ventricular function (53, 54). Yet, it has also been shown that osajin can inhibit fatty acid synthase (FASN) expression, a key enzyme for lipogenesis (55). This might enhance glycolysis, which has been associated with several processes during tissue repair and regeneration (56) as well as cardiomyocyte proliferation (13, 57). Concurrently, it has been shown that myocardial injury due to ischemia/reperfusion injury was significantly reduced by cardiac-specific PPARδ overexpression concomitant with increased myocardial glucose utilization (58).

Finally, we identified the selective α-adrenoceptor antagonist and imidazoline I_1_ receptor ligand efaroxan hydrochloride as a promotor of cardiomyocyte cell cycle progression. α-adrenoceptor and imidazoline I_1_ receptor are both expressed in cardiomyocytes and are involved in NO synthesis and intracellular calcium handling (59). Besides, efaroxan hydrochloride improved *in vivo* oral glucose tolerance (60). Yet, there is little evidence that efaroxan hydrochloride promotes proliferation. It has been reported that α2-adrenergic blockade by efaroxan hydrochloride increased primary breast tumor size and distant metastasis under non-stress conditions (61).

The efficiency of the investigated compounds to enhance cardiomyocyte cell cycle progression significantly decreased with the age of the cardiomyocytes. This phenomenon has been observed for a large number of measures to induce cardiomyocyte proliferation such as treatment with FGF1/p38 inhibitor, BIO or microRNAs as well as Meis1 deletion (24, 32, 62, 63). Thus, it will be important to elucidate the mechanisms promoting cardiomyocyte cell cycle progression utilized by the different stimuli, to determine the difference in the response of neonatal and adult cardiomyocytes to these stimuli, and to test if combinations of the different inhibitors might be able to induce robust proliferation in adult cardiomyocytes. Here, we have shown that the combinatorial approach of osajin or cardiac glycosides with PTEN or GSK3β inhibition enhances the effect on cardiomyocyte cell cycle progression. Similarly, it has been shown that the combinations of p38 MAP kinase inhibition/ PI3kinase/AKT signaling (24), GSK3β inhibition/AKT phosphorylation (4), and PPARδ signaling/GSK3β inhibition are more efficient in promoting cardiomyocyte cell cycle progression than the individual measures (14).

In the future, it will be important to determine whether the here identified drugs induce cell cycle re-entry of adult cardiomyocytes upon injury *in vivo* and to show that this contributes to improved function. Our data suggest that the positive effect of cardiac glycosides on heart function might not only increase myocardial contraction force by inhibiting the sodium-potassium pump but might also contribute to cardiac repair by inducing cardiomyocyte proliferation. Considering the toxicity of cardiac glycosides, the application of subsaturating concentrations might open new avenues for the use of cardiac glycosides, which might benefit from the combination with other pro-proliferative drugs.

## Conclusion

Considering that cardiovascular diseases represent a significant socio-economic burden affecting the pediatric as well as adult population and that currently no therapy is available to cure congenital heart disease or heart failure, the development of a cardiomyocyte proliferation screening system and the identification of novel inducers of cardiomyocyte proliferation, are potentially of great therapeutic value (64–66). Here, we identified, utilizing a novel screening system, potential compounds to promote cardiomyocyte proliferation including cardiac glycosides. Our data suggest that modulation of calcium handling and metabolism promotes cardiomyocyte proliferation and cardiac glycosides might, besides increasing myocardial contraction force, contribute to cardiac repair by inducing cardiomyocyte proliferation. The cardiomyocyte proliferation by cardiac glycosides at low concentrations enhanced by PTEN and GSK-3β inhibitors might contribute to cardiac repair by inducing cardiomyocyte proliferation. This study provides a translational perspective to investigate chemical compounds derived from the high throughput screening for their potential to improve cardiac tissue engineering approaches as well as to repair or regenerate the injured heart in pre-clinical models.

## Data availability statement

The original contributions presented in the study are included in the article/Supplementary material, further inquiries can be directed to the corresponding authors.

## Ethics statement

The animal study was reviewed and approved by the local Animal Ethics Committee Erlangen, Germany in accordance with governmental and international guidelines on animal experimentation (protocol TS-9/2016 Nephropatho).

## Author contributions

AM designed and carried out most of the experiments, analyzed most of the data, and wrote the manuscript. HR performed *in vitro* cell culture and immunostaining. NS performed immunostainings. CE provided the human iPSC-derived differentiated cardiomyocytes. RK analyzed data and revised the manuscript. FE designed experiments, analyzed data, and wrote the manuscript. All authors contributed to the article and approved the submitted version.

## Funding

This work was supported by the Emerging Fields Initiative of the Friedrich-Alexander-Universität Erlangen-Nürnberg (FAU) [EFI, CYDER: Cell Cycle in Disease and Regeneration to FE], the Alexander von Humboldt Foundation [Sofja Kovalevskaja Award to FE], and as well as the German Research Foundation (DFG) [INST 410/91-1 FUGG and IRTG 1566, PROMISE to FE].

## Conflict of interest

The authors declare that the research was conducted in the absence of any commercial or financial relationships that could be construed as a potential conflict of interest.

## Publisher's note

All claims expressed in this article are solely those of the authors and do not necessarily represent those of their affiliated organizations, or those of the publisher, the editors and the reviewers. Any product that may be evaluated in this article, or claim that may be made by its manufacturer, is not guaranteed or endorsed by the publisher.

## Supplementary material

Supplementary Figure 1 Dosage-dependent induction of DNA synthesis in neonatal cardiomyocytes. Quantitative analysis of BrdU-positive neonatal cardiomyocytes upon stimulation with the top 19 candidates at indicated concentrations. *n* = 6, mean ± SEM. Cardiomyocytes were isolated from postnatal day 3 rats, stimulated once with the individual small molecules, after 48 h BrdU was added, and after 24 h BrdU pulse-labeling the percentage of BrdU-positive cardiomyocytes was determined.

Supplementary Figure 2 Cardiac glycosides do not induce cell death in neonatal rat cardiomyocytes. Quantitative analysis of cardiomyocytes death by MTS assay (*n* = 6, mean ± SEM, ^**^: *p* < 0.01, ^*^: *p* < 0.05).

## Abbreviations

AG: Azami Green
ANOVA: analysis of variance
ara C: cytosine-D-arabinofuranoside
BIO: 6-bromoindirubin-3'-oxime
BrdU: 5-Bromo-2-deoxyuridine
cdc2: cyclin dependent kinase 1
cDNA: complementary deoxyribonucleic acid
DAPI, 4': 6'-diamidino-2-phenylindole
DMEM: Dulbecco's Modified Eagle Medium
DMSO: Dimethylsulfoxid
DNA: deoxyribonucleic acid
EB: embryoid bodies
ES: embryonic stem
FASN: fatty acid synthase
FBS: fetal bovine serum
flk1: fetal liver kinase 1
FGF1: fibroblast growth factor 1
gapdh: glyceraldehyde-3-phosphate dehydrogenase
GSK-3: glycogen synthase kinase-3
H3P: histone H3 phosphorylation on serine10
hiPSC: human induced pluripotent stem cells
IP3: inositol trisphosphate
LIF: leukemia inhibitory factor
mAG-hGem(1/110): non-functional human geminin deletion mutant fused to a monomeric version of AG
MI: myocardial infarction
myh7: myosin heavy chain 7
nkx2-5: NK2 homeobox 5
oct: octamer binding transcription factor
P3: 3-day-old
p38i: mitogen-activated protein kinase inhibitor SB203580
PBS: phosphate-buffered saline
PI3K: phosphoinositide 3-kinase
PPAR: peroxisome proliferator-activated receptor
PTEN: phosphatase and tensin homolog
RNA: ribonucleic acid
RT-PCR: reverse transcription followed by polymerase chain reaction
SEM: standard error of the mean.
