# Supplementary figures and images for: Live cell screening identifies glycosides as enhancers of cardiomyocyte cell cycle activity
Source: Front Cardiovasc Med. 2022 Sep 26;9:901396. doi: 10.3389/fcvm.2022.901396 (PMC9549374; doi:10.3389/fcvm.2022.901396)

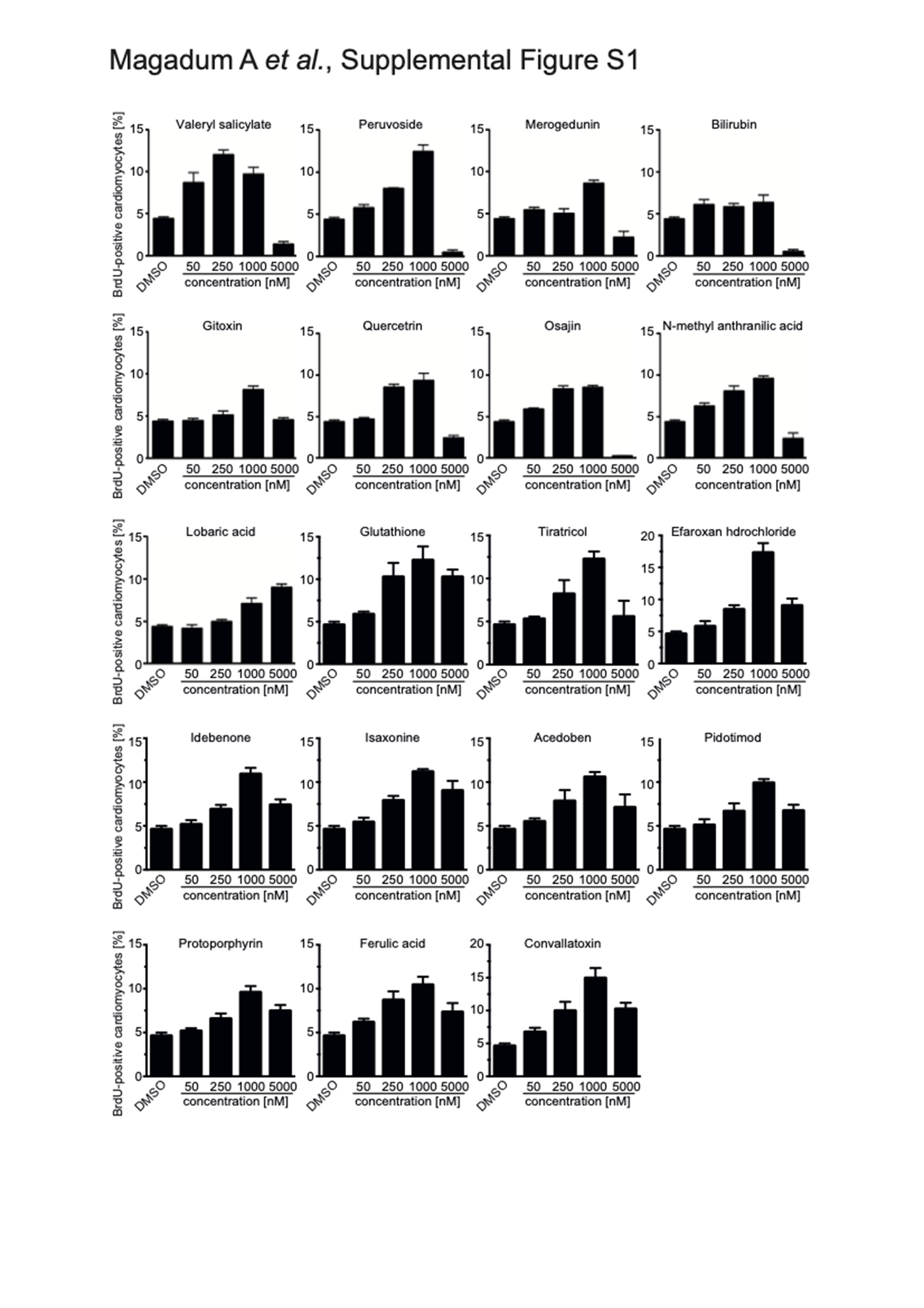

Supplement: Supplementary Figure 1 — Dosage-dependent induction of DNA synthesis in neonatal cardiomyocytes. Quantitative analysis of BrdU-positive neonatal cardiomyocytes upon stimulation with the top 19 candidates at indicated concentrations. n = 6, mean ± SEM. Cardiomyocytes were isolated from postnatal day 3 rats, stimulated once with the individual small molecules, after 48 h BrdU was added, and after 24 h BrdU pulse-labeling the percentage of BrdU-positive cardiomyocytes was determined. [file Image_1.TIFF]

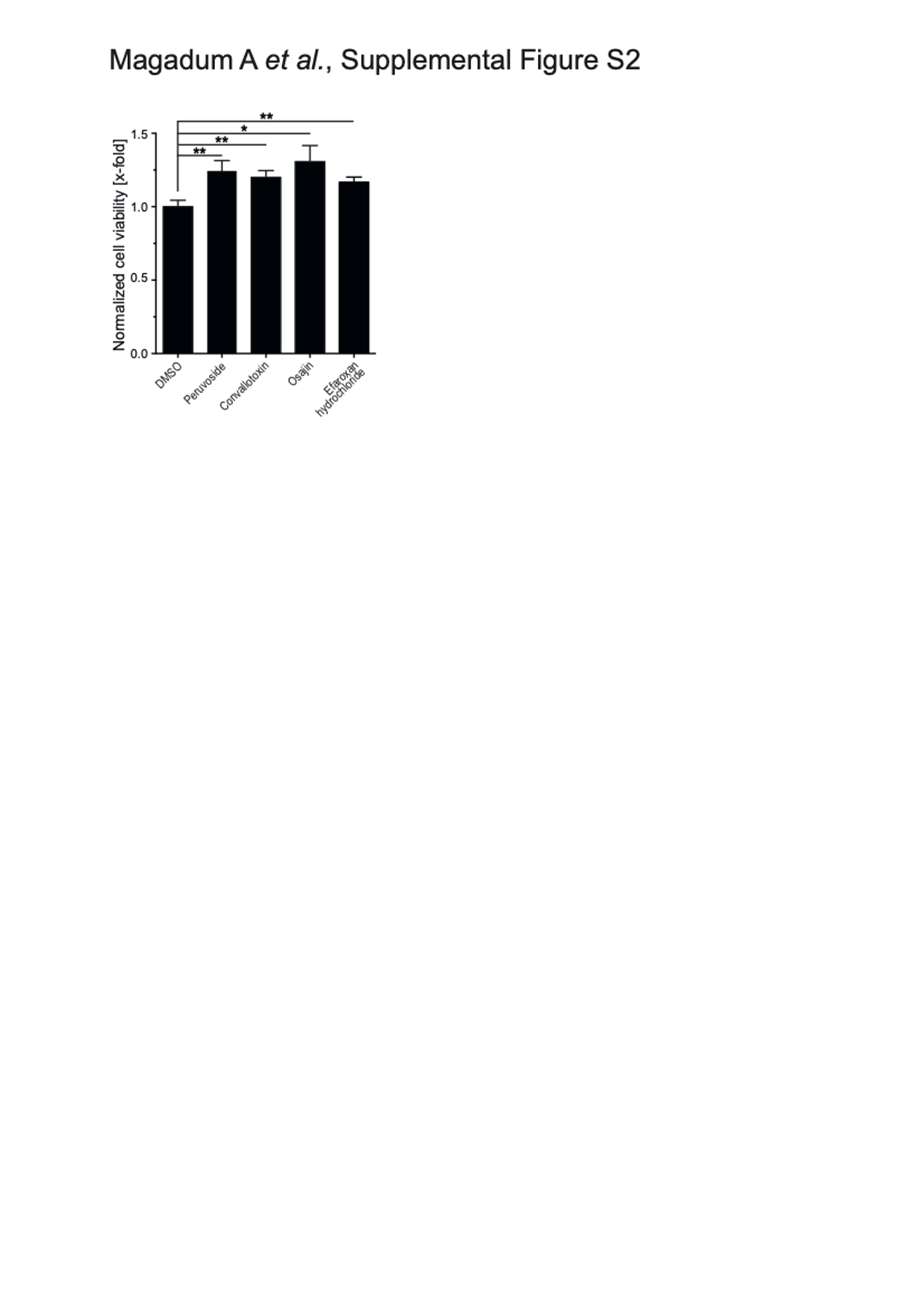

Supplement: Supplementary Figure 2 — Cardiac glycosides do not induce cell death in neonatal rat cardiomyocytes. Quantitative analysis of cardiomyocytes death by MTS assay (n = 6, mean ± SEM, **: p < 0.01, *: p < 0.05). [file Image_2.TIFF]
